# Geriatric oral health competency among dental providers

**DOI:** 10.3934/publichealth.2021054

**Published:** 2021-09-30

**Authors:** Maryam Tabrizi, Wei-Chen Lee

**Affiliations:** 1 Department of General Practice and Dental Public Health, School of Dentistry, The University of Texas Health Science Center at Houston, Houston, TX, United States; 2 Department of Internal Medicine, The University of Texas Medical Branch, Galveston, TX, United States

**Keywords:** geriatric population, oral health, dental provider, health literacy

## Abstract

**Background:**

Geriatrics as an educational topic has been a high priority in current health care. The innovative Age-Friendly health system with the 4Ms structure (what Matters most, Medication, Mentation, Mobility) needs to be integrated into oral health and dental services training. The purpose of this study is to respond to one question: are the graduating general dentists trained and prepared to treat medically vulnerable elderly in communities?

**Methods:**

All pre-doctorate dental students from first year to fourth year were invited to voluntarily respond to an online survey provided on Qualtrics. The survey provided examples of two broken molar teeth that need extraction. First, students were asked how comfortable they felt extracting the two molars based on the x-rays. Then, the question was repeated to evaluate if they felt comfortable with extracting the teeth in a patient with one chronic condition and related medication(s). Finally, the students were again questioned whether they feel comfortable to provide the same service to medically vulnerable patients with multiple health conditions and polypharmacy.

**Results:**

The majority of students who participated in this study said they were comfortable with extracting the teeth of patients without any chronic condition. However, many more chose to refer medically vulnerable patients with multiple chronic conditions and polypharmacy to a specialist.

**Conclusions:**

Dental education in many U.S. dental schools may provide adequate education and create competent general dentists. Yet, the competency and confidence required for dentists to be able to treat older adults with multiple health conditions and using prescribed or over-the-counter medication is insufficient.

## 1. Introduction

By 2030, the number of adults in the USA over the age of 65 will increase more than 30% (approx. 56 million in 2020 to 74 million in 2030) [1]. The scientific literature also shows that by 2030, over 22 million older Americans will need specialized geriatrician care [2]. Due to the high demand, the national shortage of geriatricians will be close to 27,000 full time equivalent (FTE) positions in 2025 [3]. The degree of shortage in each region is shown in Table 1. In the South region, currently there is a demand for an additional 6,900 geriatricians and this shortage will continue beyond 2025. The impact of the shortage will be even more severe in rural/underserved areas.

**Table 1.** Baseline and projected geriatrician supply and demand by region, 2013 and 2025 [3].

Region	2013 Baseline Estimates (FTEs)			2025 Projections (FTEs)		
	Supply	Demand ^a^	Difference ^b^	Supply	Demand	Difference ^b^
Northeast	1,050	4,920	−3,870	1,490	4,380	−2,890
Midwest	650	4,920	−4,270	1,040	4,470	−3,430
South	1,150	8,050	−6,900	2,150	8,280	−6,130
West	740	5,050	−4,310	1,540	16,070	−14,530

Note: The South region has 16 states, including Texas. ^a^ Baseline supply and demand are not in equilibrium in the regions because regional demands were estimated by prorating national geriatrician and demand based on regional population characteristics (e.g., age, sex, household income, insurance status, health status, etc.). ^b^ Difference = (supply – demand); a negative difference reflects a shortage while a positive difference indicates a surplus.

Along with the increasing number of older adults is the prevalence of multiple chronic conditions [4]. Polypharmacy patients often take combination of drugs that have additive anticholinergic effects that increases risk of developing xerostomia and the subsequent oral health complications. Oral health problems in older adults represent a silent, serious public health issue. Nearly all adults (96%) aged 65 years and older have had a cavity and about 2 in 3 (68%) have gum diseases [5]. Lack of adequate education and training among dental and non-dental providers and lack of Medicare funding for oral health place older adults at a higher risk for dental and physical complications.

Despite oral problems in American older adults, few studies have tackled the topic of dental education and the competency of incoming graduates to care for older adults with physical and mental vulnerabilities. In a 2017 study, a survey from 56 of all 67 U.S. dental schools showed that geriatric dentistry was taught in all responding schools. More than a half (62.5%) were teaching it as an independent course and only five schools (8.9%) were teaching it as occasional lectures [6]. This result represents a measurable improvement since 2013, when only 22.6% of surveyed dental schools offered clinical training specifically related to older adults [7]. However, the measurements between the two studies are not consistent and no other evaluation has been conducted in U.S. dental schools.

Geriatric oral health requires interprofessional education and collaboration across all health disciplines. Our previous study found that, when students from four disciplines worked together during a simulated health crisis, they learned to communicate adequately and provide patient-centered care which may ultimately lead to better clinical outcomes [8]. This study is designed to follow the source of the issue at its core. The research question was: do dental students get adequate education on geriatric dentistry to be competent enough to treat medically vulnerable elderly?

## 2. Materials and methods

### 2.1. Study participants and setting

The study was performed in one school of dentistry where students receive formal dental education and training over four years. The study recruited all levels of dental students (DS1-DS4) in 2018 and 2019 and surveyed their knowledge and competence every year until 2020.

The study was approved by the Institutional Review Board. Participation in this study was completely voluntary and anonymous. The study did not follow any specific cohort and participants had the right to join/withdraw at any time during the study period.

### 2.2. Data collection and analysis

**Figure 1.**
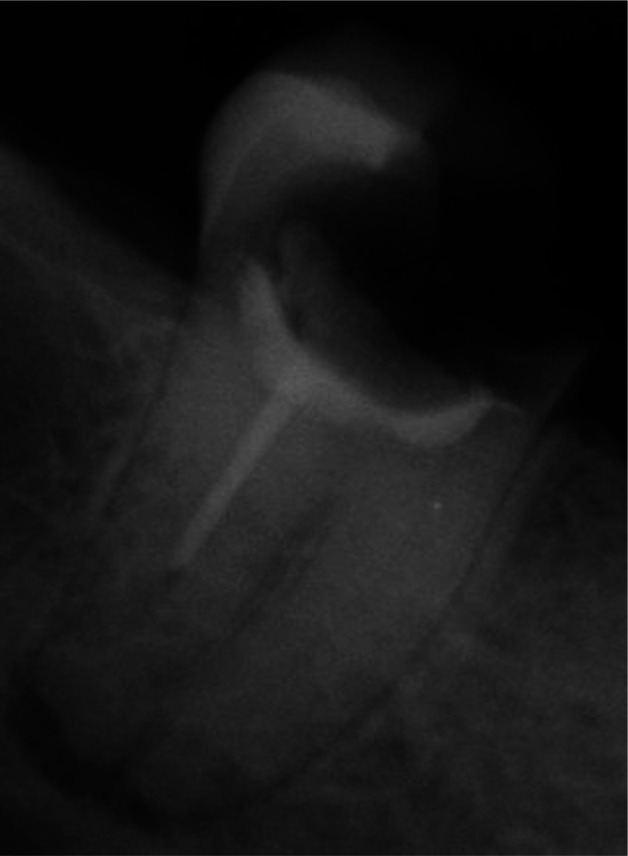
The first image to test students' literacy.

**Figure 2.**
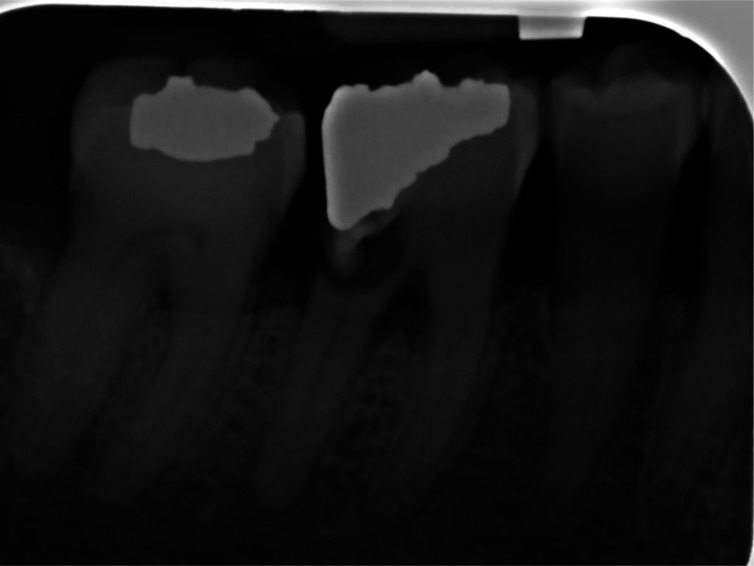
The second image to test students' literacy.

The survey's questions were based on two radiographs of broken teeth that needed surgical extraction (Figure 1 and Figure 2) and both radiographs were presented to the students via the Qualtrics survey tool. For each radiograph, students were given the background information and health status of the patient. Next, the participants were asked four questions to test their extraction-related decision making:

a) Would you extract this tooth (*Yes* or *No*)?

b) What is the level of difficulty to extract the tooth as shown on the x-ray (four levels from *Very Easy* to *Very Difficult*)?

c) What is the level of difficulty to extract the tooth as shown on the x-ray if the patient indicated controlled or uncontrolled diabetes (four levels from *Very Easy* to *Very Difficult*)?

d) What is the level of difficulty to extract the tooth as shown on the x-ray if the patient indicated two or more health conditions among diabetes, cardiovascular diseases, Alzheimer's disease, dementia, and other physical limitations (four levels from *Very Easy* to *Very Difficult*)?

The descriptive analysis was conducted to assess the background of participants. Participants who were residents or faculty were excluded from the final sample. Next, binary analysis was conducted to examine the relationship between the study year of the student and the reported decision and level of difficulty. The first question has a binary outcome, so we used the Fisher's Exact Test to yield significance results. The second to fourth questions are categorical with four responses. We used both Kruskal-Wallis rank sum test and Turkey-Kramer-Nemenyi all-pairs test with Turkey-Dist approximation to yield significance results. Four questions were the same for both radiographs and we simply repeated the analyses twice. All analyses were carried out by using R statistical software package. A two-sided *p*-value less than 0.05 was considered as statistically significant.

## 3. Results

For the questions related to the first image, we received 10 responses from the first-year student (i.e., DDS1), 26 from DDS2, 45 from DDS3, and 80 from DDS4. For the questions related to the second image, we received 9 responses from the DDS1 student, 20 from DDS2, 25 from DDS3, and 49 from DDS4. Because we did not have enough students who consistently participated in the study from 2018 to 2020, we complied all the responses and divided them by the calendar year when we released the survey. The final study sample included 10 DDS1 students, 24 DDS2, 32 DDS3, and 58 DDS4. Table 2 shows the distribution of responses collected by students' self-reported school years. By 2020, most students had started their 4^th^ year and there was no student in the first to third years. Over three years, the total number of valid responses is 124.

**Table 2.** School year of participants from 2018 to 2020.

	2018 (n = 75)	2019 (n = 37)	2020 (n = 12)
DDS1 (n = 10)	10 (13.3%)	0 (0.0%)	0 (0.0%)
DDS2 (n = 24)	21 (28.0%)	3 (8.1%)	0 (0.0%)
DDS3 (n = 32)	15 (20.0%)	17 (45.9%)	0 (0.0%)
DDS4 (n = 58)	29 (38.7%)	17 (45.9%)	12 (100.0%)

**Table 3.** Relationship between the year of students and their respective responses.

	DDS1	DDS2	DDS3	DDS4
**I. First Image**				
1) Extract tooth*				
a. Yes	6 (60.0%)	22 (95.7%)	28 (96.6%)	46 (83.6%)
b. No	4 (40.0%)	1 (4.3%)	1 (3.4%)	9 (16.4%)
2) Only Image				
a. Very Easy	1 (10.0%)	2 (9.1%)	2 (6.9%)	6 (10.9%)
b. Easy	1 (10.0%)	6 (27.3%)	14 (48.3%)	24 (43.6%)
c. Difficult	7 (70.0%)	14 (63.6%)	12 (41.1%)	19 (34.5%)
d. Very Difficult	1 (10.0%)	0 (0.0%)	1 (4.5%)	6 (10.9%)
3) With Diabetes				
a. Very Easy	0 (0.0%)	0 (0.0%)	2 (7.4%)	2 (3.6%)
b. Easy	2 (20.0%)	3 (13.0%)	8 (29.6%)	17 (30.9%)
c. Difficult	2 (20.0%)	17 (73.9%)	13 (48.1%)	27 (49.1%)
d. Very Difficult	6 (60.0%)	3 (13.0%)	4 (14.8%)	9 (16.4%)
4) With ≥2 Conditions				
a. Very Easy	0 (0.0%)	0 (0.0%)	1 (3.3%)	1 (1.9%)
b. Easy	2 (22.2%)	3 (12.5%)	3 (10.0%)	8 (14.8%)
c. Difficult	4 (44.4%)	6 (25.0%)	9 (30.0%)	20 (37.0%)
d. Very Difficult	3 (33.3%)	15 (62.5%)	17 (56.7%)	25 (46.3%)
**II. Second Image**				
1) Extract tooth*				
a. Yes	9 (100.0%)	17 (85.0%)	19 (76.0%)	44 (89.8%)
b. No	0 (0.0%)	3 (15.0%)	6 (24.0%)	5 (10.2%)
2) Only Image				
a. Very Easy	0 (0.0%)	4 (19.0%)	1 (4.2%)	8 (16.7%)
b. Easy	6 (75.0%)	10 (47.6%)	15 (62.5%)	28 (58.3%)
c. Difficult	1 (12.5%)	5 (23.8%)	8 (33.3%)	10 (20.8%)
d. Very Difficult	1 (12.5%)	2 (9.5%)	0 (0.0%)	2 (4.2%)
3) With Diabetes				
a. Very Easy	0 (0.0%)	1 (4.8%)	1 (4.2%)	2 (4.1%)
b. Easy	5 (62.5%)	6 (28.6%)	10 (41.7%)	24 (49.0%)
c. Difficult	2 (25.0%)	10 (47.6%)	10 (41.7%)	21 (42.9%)
d. Very Difficult	1 (12.5%)	4 (19.0%)	3 (12.5%)	2 (4.1%)
4) With ≥2 Conditions				
a. Very Easy	0 (0.0%)	1 (4.8%)	0 (0.0%)	1 (2.0%)
b. Easy	4 (50.0%)	2 (9.5%)	7 (29.2%)	12 (24.5%)
c. Difficult	1 (12.5%)	8 (38.1%)	8 (33.3%)	27 (55.1%)
d. Very Difficult	3 (37.5%)	10 (47.6%)	9 (37.5%)	9 (18.4%)

Note: *: *p* < 0.05.

Table 3 describes the relationship between the year of study of students and their answers to four questions per radiograph. The relationship between year of student and agreement to extract the tooth based on the first and second radiographs was statistically significant. We did not find any significant results for the other questions.

Overall, dental student at the advanced stage of dental education considered themselves competent primary care oral health providers but not so competent in treating the medically vulnerable elderly. For the first radiograph, over 50% of DDS4 students found extracting the tooth easy or very easy. However, when they were told that this patient had diabetes or more than two comorbid conditions, the percentage dropped below 50%. Similarly, the percentage of DDS4 students who found extracting the tooth easy or very easy dropped after learning of the patient's chronic conditions.

## 4. Discussion

The study sought to evaluate the dental education advancement of dental students as well as their application of dental knowledge and service as primary oral health providers for individuals with complex overall health conditions and medication use. To examine the gap in dental education in terms of geriatric oral health, a convenience sample of dental students was recruited to complete the survey. Before the cases were presented with chronic conditions, we saw the statistically significant differences in the association between the grade of students and the confidence of students in extracting the tooth. After we presented the elderly cases with chronic conditions, there is no significance across four years. Due to a relatively small sample of respondents, the level of generalizability may be restricted, and the study result should be interpreted with cautions. However, our study indeed demonstrated that much of this group of students after four years of dental education did not feel confident to extract teeth in the elderly with complex health conditions and multiple medications. Namely, the inadequate training to care for geriatric populations coupled with the understanding of challenging cases reduce students' confidence in extracting teeth. Most of the students did not believe they had received adequate education and clinical training to provide invasive dental services such as complicated exactions that they would have performed in healthier patients.

Although most dental schools in the United States offer geriatric education, the instruction can range from a lecture or two threaded within different courses and under different titles, to stand-alone curricula including both didactic and clinical teaching. The students in our program had threads of geriatric education in different courses and one geriatric rotation session in the second year. The session is a four-hour rotation in nursing homes to expose students to the oral conditions and the need for geriatric oral health care in long term care facilities. Nevertheless, there was no treatment or clinical education involved in these rotations. To address these educational gaps in pre-doctoral dental education for vulnerable populations, we discuss six strategies as below.

*1) Enhance Training in Dental Schools*. The Council on Dental Accreditation (CODA) defines people with special needs as individuals with developmental disabilities, cognitive impairment, complex medical problems, significant physical limitations, and the vulnerable elderly [9]. The National Council on Disability (NCD) reported that more than 50% of dental schools did not equip their students with competency to treat medically compromised individuals with special needs. Therefore, the CODA recently voted to require dental schools to train their students in managing treatment of patients with disabilities. Adequate training should also incorporate the evidence-based 4Ms model (what Matters most, Medication, Mentation, Mobility) so that dental professionals are aware of the needs of older adults^.^[10].

*2) Create an Adequate Workforce Through Career Awards*. The Health Resources and Services Administration (HRSA) has recognized the lack of oral health care in existing health systems and has taken the initiative to improve oral health. For the first time, HRSA provides a Geriatric Academic Career Award (GACA) dedicated to enhancement of the workforce in oral health in accredited dental schools. It provides clinical training in geriatrics, including the training of interprofessional teams of health care professionals [11]. The GACA program also supports the career development of junior faculty in geriatrics at accredited schools of allopathic medicine, osteopathic medicine, nursing, social work, psychology, dentistry, pharmacy, or allied health as academic geriatrics specialists.

*3) Integrate Geriatric Care into Dental Care*. The 2018 America Dental Association survey showed that86 percent of Americans believe that oral health is very important to their overall health [12]. Yet, only 15 percent rated their current oral health as excellent. After recognizing the complex needs for dental services among geriatric populations, both American Dental Education Association (ADA) and American Dental Education Association (ADEA) have developed clinical guides for oral health professionals [13],[14]. The guide will support professionals to better evaluate and diagnose dental problems, link oral health to chronic conditions, and provide treatment to enhance older adults' quality of life.

*4) Reimburse Oral Care for The Elderly*. Despite the fact that oral health is essential to overall health, the Centers for Medicare and Medicaid Services (CMS) have not included oral health in Medicare. Beneficiaries must pay for routine services directly out-of-pocket, or rely on dental coverage through private plans or Medicaid. Due to this lack of dental coverage, more than half of all beneficiaries do not use any dental services in a given year [15]. After witnessing the shortage of healthcare workforce during the pandemic, a study suggested that CMS should strategically plan for and support the creation of a qualified workforce through expanding its coverage [16].

*5) Strengthen the Role of Public Health*. Oral health is much more than just heathy teeth. The American Public Health Association (APHA) has acknowledged the lack of oral health care for older adults and have pledged to help strengthen the support, advocacy, and policies for greater health care in older adults. In its “20204 Adult dental care in Medicaid and Medicare” statement, the APHA recommends that state government agencies provide comprehensive dental benefits for Medicaid beneficiaries [17]. The APHA also calls on state lawmakers to allow for and expand alternative models (e.g., tele-dentistry) of the dental workforce and provide sufficient dental care reimbursement to ensure that providers want to participate.

*6) Adopt New Delivery Models*. Increasing access to telehealth for older adults and caregivers is important, a lesson learned from the pandemic [12]. Older adults who are less dependent may have a higher acceptance of technology. Teledentistry has been approved in most States, including Texas. However, how much teledentistry can open access, decrease disparity, and address inequities for the diverse population of older adults in the U.S is unknown. Two recent systematic reviews have compared the applications of teledentistry, yet neither was particularly focused on geriatric oral health [18],[19]. Therefore, more studies are needed to examine the effectiveness of teledentistry on health outcomes in geriatric populations. Likewise, investigating the provider's perspective on communicating with patients through technology would inform the continued upgrading of technology and implementation of appropriate training.

## 5. Conclusions

Current dental training is clearly inadequate to train students to extract teeth from geriatric populations with multiple chronic conditions. Facing the increased number of older adults, new and innovative strategies to improve the education of the dental workforce are recommended to enhance older adults' access to appropriate dental care. Studies to examine the gaps in dental training should also be continued in the future.
